# Gas-Phase Deposition of Ultrathin Aluminium Oxide Films on Nanoparticles at Ambient Conditions

**DOI:** 10.3390/ma8031249

**Published:** 2015-03-19

**Authors:** David Valdesueiro, Gabrie M. H. Meesters, Michiel T. Kreutzer, J. Ruud van Ommen

**Affiliations:** Department of Chemical Engineering, Delft University of Technology, 2628 BL Delft, The Netherlands; E-Mails: d.valdesueiro@tudelft.nl (D.V.); G.M.H.Meesters@tudelft.nl (G.M.H.M.); M.T.Kreutzer@tudelft.nl (M.T.K.)

**Keywords:** atomic layer deposition (ALD), coating, nanoparticles, aluminium oxide, thin films, fluidized bed reactor, ambient conditions, room temperature, atmospheric pressure

## Abstract

We have deposited aluminium oxide films by atomic layer deposition on titanium oxide nanoparticles in a fluidized bed reactor at 27 ± 3 °C and atmospheric pressure. Working at room temperature allows the coating of heat-sensitive materials, while working at atmospheric pressure would simplify the scale-up of this process. We performed 4, 7 and 15 cycles by dosing a predefined amount of precursors, *i.e.*, trimethyl aluminium and water. We obtained a growth per cycle of 0.14–0.15 nm determined by transmission electron microscopy (TEM), similar to atomic layer deposition (ALD) experiments at a few millibars and ~180 °C. We also increased the amount of precursors dosed by a factor of 2, 4 and 6 compared to the base case, maintaining the same purging time. The growth per cycle (GPC) increased, although not linearly, with the dosing time. In addition, we performed an experiment at 170 °C and 1 bar using the dosing times increased by factor 6, and obtained a growth per cycle of 0.16 nm. These results were verified with elemental analysis, which showed a good agreement with the results from TEM pictures. Thermal gravimetric analysis (TGA) showed a negligible amount of unreacted molecules inside the alumina films. Overall, the dosage of the precursors is crucial to control precisely the growth of the alumina films at atmospheric pressure and room temperature. Dosing excess precursor induces a chemical vapour deposition type of growth due to the physisorption of molecules on the particles, but this can be avoided by working at high temperatures.

## 1. Introduction

The production of core-shell nanoparticles using atomic layer deposition (ALD) in a fluidized bed reactor (FBR) is an attractive technology because of the good mixing between gas and solids, and the possibility to process large amounts of solids up to industrial scale [1,2,3,4]. For instance, aluminium oxide (Al_2_O_3_) coating obtained by ALD on particles in a FBR was applied as a passivating barrier against oxidation, as a gas barrier on sensors, and in the production of catalysts, amongst other applications [5,6,7,8,9,10]. Nanoparticles are widely used in diverse fields such as catalysis, medicine, energy conversion and storage [11,12,13,14]. In addition to the large specific surface area, surface modification can introduce additional functionalities to improve these nanomaterials. Atomic layer deposition, which was developed for the semiconductor industry [15,16], appears as an enabling coating technique to either protect or activate the surface of substrates with a precise control over the amount of material deposited [11,17,18,19,20,21]. The ALD layer-by-layer growth mechanism relies on two alternating gas phase reactions with a purging step in between, using with an inert gas to remove the unreacted molecules of precursor. These reactions can be repeated a certain number of times to deposit conformal and pin-hole free thin films with a precision down to atomic scale. In addition, the absence of solvent in ALD processes reduces the waste generated when considering industrial scale production [3]. These features establish the ALD in a FBR as a suitable technique to produce core-shell nanoparticles with tailored functionalities.

The deposition of Al_2_O_3_ is typically done at low pressure (~1 mbar or lower) and elevated temperatures (~180 °C) to enable the evaporation of the excess molecules of precursors, and in this manner ensure atomic growth of the films by an efficient removal of the excess precursors [22]. The physisorption of unreacted molecules at ambient conditions would result in undesired parasitic CVD-type (chemical vapour deposition) of reactions [23,24]. ALD of alumina has been studied at different reactor temperatures ranging from 33 °C to ~180 °C, and pressures, from 10^−5^ bar to 1 bar, over a diverse variety of powders. To our knowledge, alumina ALD at both low temperature and atmospheric pressure has not yet been investigated, neither on flat substrates nor on particles. The growth per cycle (GPC) is used to characterize the coating experiments [25,26]. ALD at pressures of a few millibars and ~180 °C provides alumina layers with a growth between 0.1 and 0.2 nm per cycle [27,28,29,30,31,32]. At atmospheric pressure and 160 °C Al_2_O_3_, ALD revealed the possibility of depositing few-nanometre films with slightly larger growth per cycle [33]. Alumina ALD performed at 10^−5^ bar and 33 °C gave a GPC of 0.3 nm [34,35]. With regards to flat substrates, more studies were reported either at room temperature and low pressure [36,37,38], or at atmospheric pressure and a temperature above 100 °C [39,40] showing an influence of these variables on the GPC, which is higher than at lower pressure and higher temperature. These conditions can be improved in two different ways. First, working at room temperature opens the possibility to apply ALD on heat-sensitive materials [41]. Secondly, using atmospheric pressure would facilitate the scale-up towards the industrial production by easing the handling of the powder and reducing the complexity of the equipment required. For these reasons, we study the deposition of alumina on nanoparticles at room temperature and atmospheric pressure.

This experimental paper describes the aluminium oxide ALD in a FBR at 1 bar and 27 ± 3 °C, using TiO_2_ P25 nanoparticles as the support. The objective of the paper is to understand whether conformal alumina films can be deposited at ambient conditions, supressing the physisorption of the excess precursor molecules by a careful dosage of the precursors. For that, we performed three sets of experiments. In the first one, we examined whether we can achieve a growth per cycle that is similar to that reported in the literature at lower pressure and higher temperature. In the second set, we determined if the precursor would physisorb on the surface of the particles if the dosing of precursor is extended, at constant concentration, thus increasing the excess of precursor relative to the amount of reactive sites inside the reactor. In the third set, we investigated the GPC of the alumina films at 1 bar and 170 °C when both precursors are fed in excess. In this work, we consider the dosed amount of the precursors as a crucial factor to avoid the CVD-type growth expected at ambient conditions, particularly in the case of water [39,42,43]. These molecules would react in the subsequent reaction, resulting in thicker and non-uniform alumina films. We try to avoid the accumulation of unreacted molecules with an accurate delivery of the precursors. That would allow the deposition of ultrathin films at room conditions, while maintaining the control over the properties of the films.

## 2. Experimental Section

### 2.1. Experimental Setup

Experiments are carried out in a fluidized bed reactor similar to the one described by Beetstra *et al.* [33], which is composed by a vertical glass column of 26 mm internal diameter and 500 mm height, placed on a single motor Paja PTL 40/40-24 vertical vibration table to assist the fluidization [2]. The vibration table is operated at 35 Hz, and provides a vibration amplitude of 2 mm to the column. An infrared lamp placed parallel to the column, and a type-K thermocouple inserted in the column, are used to control the bed temperature. The gas is introduced to the column through a stainless steel SIKA-R 20 AX distributor plate of sintered particles with a pore size of 37 μm, to achieve a homogenous flow of gas through the full cross-section of the column. An identical distributor plate is placed on top of the column to prevent nanoparticles from leaving the column. Although the pore size of the distributor plates is several orders of magnitude larger than the particle size, the risk of losing particles is small since they do not fluidize as individual particles, but as agglomerates of 200–300 μm [2]. These agglomerates, which have a void fraction of 98%–99% [44], demonstrate a dynamic behavior during fluidization [45]. This means that the agglomerates break and recombine constantly as a consequence of the collisions between solids. The dynamic behavior and large porosity of the agglomerates ensure that the precursor molecules reach the whole surface area of the individual particles, even though they exist as agglomerates.

Aeroxide P25 titanium oxide (TiO_2_) particles from Evonik Industries (Hanau, Germany) are used as the substrate for the coating experiments. This powder has a Sauter mean diameter (*d*_3,2_) of 32.7 nm (Supplementary Information S1), a specific surface area of 52.4 m^2^/g, and a surface concentration of hydroxyl groups of 5.0 OH/nm^2^ [46]. Semiconductor grade TMA (trimethyl aluminium) was provided by Akzo Nobel HPMO (Amersfoort, The Netherlands) in a 600 mL WW-600 stainless steel bubbler, which is kept at 30 °C during the coating experiments. The second precursor, demineralized water, is kept in a similar bubbler. Pressurized nitrogen grade 5.0 is provided to the column as the carrier gas; no pump is present after the column. The column is always kept at atmospheric pressure. During start-up of an experiment, we first used nitrogen to drive away the air before starting the coating. The off-gas of the fluidized bed was led through a rack of five washing bubblers filled with Kaydol oil to remove possible traces of unreacted precursors and the products of the reactions.

The precursor bubblers, the fluidized bed reactor and the washing bubblers are placed inside a nitrogen-blanketing cabinet as a TMA safety measure. The cabinet is operated at an O_2_ concentration below 6%. For each experiment, 2.00 g of TiO_2_ powder were placed inside the column. A flow of 0.4 L/min of nitrogen, which corresponds to a superficial gas velocity of 1.26 cm/s, was applied to fluidize the powder.

### 2.2. Design of the Experiments

To calculate the precursor dosing times, we estimated the total amount of active sites in the bed of particles, *i.e.*, hydroxyl groups. This amount is calculated with the surface area of the TiO_2_ (52.4 m^2^/g), the mass of powder placed inside the column (2.00 g), and the surface concentration of hydroxyl groups (5.0 OH/nm^2^) [46]. For 2.00 g of powder, we have an initial amount of 8.7 × 10^−4^ mol of OH. To calculate the amount of TMA dosed to the reactor, we assumed that at 30 °C, which is the TMA bubbler temperature, TMA forms dimers [47,48,49,50]. We estimated that the saturation of the nitrogen bubbles with TMA inside the bubbler, using the model proposed by Mayer *et al.* [51], is about 50% when the bubbler is filled with TMA up to the secure level. In addition, we assumed that the entire particle surface area is exposed to TMA in the gas phase (*i.e.*, no “dead zones”) because fluidized bed reactors provide intense contact between precursors and solids [52,53]. Based on these assumptions, and using the vapour pressure of TMA and the ideal gas law, we obtained a flow of TMA to the reactor of 4.8 × 10^−4^ mol/min, which translates into an ideal dosing time of 1.7 min for TMA. To account for the assumptions used in the theoretical estimation, and ensure that the surface of the powder inside the column is fully saturated, we doubled the resulting dosage time for TMA and water. As a result, a significant fraction of the TMA leaves the reactor without reacting. This inefficient use of TMA is an acceptable penalty for the objective of this paper, *i.e.*, to find out how much excess of precursor can be tolerated close to physisorbing conditions.

From the calculation above, we obtained a final dosing time of 3.5 min for TMA, and 2.5 min for water. After dosing each precursor to the reactor, we purge the system with N_2_ for 10 min (which corresponds to 13 residence times), establishing a feeding sequence of TMA–N_2_–H_2_O–N_2_. With these dosing times (3.5–10–2.5–10 min), which we will consider as the base case situation, we performed 4, 7, and 15 cycles. In the second set of experiments, we studied the deposition of alumina when increasing the dosing times of precursors and keeping the same purging time of nitrogen. For this study, we perform 7 cycles at three different dosing times: 2 times (7–10–5–10 min), 4 times (14–10–10–10 min), and 6 times (21–10–15–10 min) larger than in the base case study. In addition, we performed 5 cycles at 1 bar and 170 °C with dosing times of 21–10–15–10 min, to investigate whether we can avoid the physisorption of the molecules in excess and gain the self-limitation of the ALD reactions, by increasing the reactor temperature.

### 2.3. Characterization of the Coating

To characterize the deposition process, we determined the thickness of the alumina film by both TEM and elemental analysis, and divided it by the number of cycles to calculate the growth per cycle. For these measurements, we used TEM (transmission electron microscopy) with EDX (energy dispersive X-ray spectroscopy), done with a Tecnai TF20 (FEI, Hillsboro, OR, USA), which provides direct observation of the thickness of the coating δ_TEM_. Elemental analysis was carried out using ICP-OES (Induced Couple Plasma Optical Emission Spectroscopy, performed with a PerkinElmer Optima 5300, PerkinElmer, Waltham, MA, USA), which provides the mass fraction of aluminium in the coated sample *x*_Al_. To determine *x*_Al_ in the samples, we first destructed an amount of 50 mg of the coated powder in a solution prepared with 1.5 mL 65% HNO_3_ + 4.5 mL 30% HCl + 1 mL 40% HF using the microwave. After destruction, the samples were diluted to 50 mL with MQ (milli-Q) water. The samples were then analyzed with ICP-OES to determine the mass fraction of aluminium in the samples. With this value, we calculated, using Equation (1), the corresponding volume of aluminium oxide in each particle $V_{{\text{Al}}_{2}{\text{O}}_{3}}^{1P}$, assuming spherical TiO_2_particles with a diameter *d*_3,2_ and density ${\text{ρ}}_{{\text{TiO}}_{2}}$, using the molecular mass of alumina $M_{{\text{Al}}_{2}{\text{O}}_{3}}$ and aluminium $M_{\text{Al}}$, a factor of ½ because there are 2 moles of Al in one mole of Al_2_O_3_, and a density of the film of aluminium oxide ${\text{ρ}}_{{\text{Al}}_{2}{\text{O}}_{3}}$ of 2500 kg/m^3^, equal to the value reported by Groner *et al.* [36] for Al_2_O_3_ ALD at room temperature. We presume that the density of alumina deposited by ALD on particles would be smaller than the one deposited on flat substrates, due to the discontinuities between the facets of the particles.

(1) $$V_{{\text{Al}}_{2}{\text{O}}_{3}}^{1\text{P}}=\frac{x_{\text{Al}}}{1-\frac{M_{{\text{Al}}_{2}{\text{O}}_{3}}}{2\cdot M_{\text{Al}}}\cdot x_{\text{Al}}}\cdot \frac{M_{{\text{Al}}_{2}{\text{O}}_{3}}}{2\cdot M_{\text{Al}}}\cdot \frac{{\text{ρ}}_{{\text{TiO}}_{2}}}{{\text{ρ}}_{{\text{Al}}_{2}{\text{O}}_{3}}}\cdot \frac{\text{π}}{6}\cdot d_{3,2}^{3}$$

Then, we used Equation (2) to calculate the thickness of the film ${\text{δ}}_{\text{ICP}}$ based on the volume of alumina per particle determined with Equation (1). Assuming spherical particles, we calculated the thickness of the alumina film as the difference of the volume between the core-shell particle, and the uncoated TiO_2_ P25 particle. Using Equations (1) and (2), we account for the curvature of the particles to estimate of the thickness of the alumina film ${\text{δ}}_{\text{ICP}}$:

(2) $${\text{δ}}_{\text{ICP}}=\frac{\sqrt[{3}]{\frac{6}{\text{π}}\cdot V_{{\text{Al}}_{2}{\text{O}}_{3}}^{1P}+d_{3,2}^{3}}-d_{3,2}}{2}$$

In the calculation of the film thickness, we used average values for particle size, mass fraction of aluminium and density of the aluminium oxide; however, that implies an inaccuracy due to the experimental error. To account for that, we performed the propagation of the uncertainty (Supplementary Information S2), using a Sauter mean diameter $d_{3,2}$ of 32.7 ± 3.3 nm (Supplementary Information S1). The errors correspond to 10% of the measurement of the diameter $\text{∆}d_{3,2}$, 3% of the mass fraction value from ICP-OES $\text{∆}x_{\text{Al}}$, and 200 kg/m^3^ for the density of alumina $\text{∆}{\text{ρ}}_{{\text{Al}}_{2}{\text{O}}_{3}}$, to obtain the interval of confidence for the film thickness ${\text{δ}}_{\text{ICP}}$.

## 3. Results and Discussion

This section is divided in four parts: (i) experiments performed with the base case dosing times of the precursors; (ii) experiments done to study the self-limitation of the reactions when feeding excess of precursors at 1 bar and 25 °C; and (iii) at 1 bar and 170 °C, and (iv) a study of density of the alumina films using BET (Brunauer-Emmett-Teller) N_2_-adsorption and thermal gravimetric analysis (TGA) (Mettler Toledo TGA/SDTA 851 e, Greifensee, Switzerland).

### 3.1. Base Case Coating Experiments

We performed 4, 7, and 15 ALD cycles with a dosage sequence of 3.5–10–2.5–10 min, of TMA, nitrogen, water, and nitrogen, respectively, using a flow of 0.4 L/min of N_2_. We measured the film thickness of a significant number of points and particles (Supplementary Information S3 and S4) for a proper analysis of the coated samples. Measuring the film thickness after 4 cycles is complex since each cycle deposits about 30% of a monolayer as consequence of the steric hindrance between the alkyl ligands, and the density of active sites on the surface of the particles [54]. Such thin films of around 0.6 nm could be deceiving if the particles are not properly focused in the TEM images. In addition, we calculated the growth per cycle after dividing the thickness by the number of ALD cycles, plotted the histograms, and calculated the mean value and the standard deviation of all the measurements. With this characterization we evaluated the thickness of the film, the deposition rate, and conformality of the alumina films.

**Figure 1:**
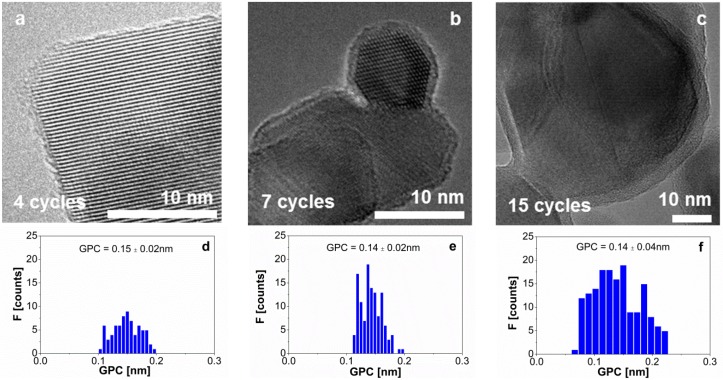
TEM images of the alumina film on TiO_2_ nanoparticles for a dosing sequence of 3.5–10–2.5–10 min after (**a**) 4 ALD cycles; (**b**) 7 ALD cycles; and (**c**) 15 ALD cycles. (**d**) Distribution of the growth per cycle from the TEM pictures after 4 ALD cycles; (**e**) after 7 ALD cycles; and (**f**) after 15 ALD cycles. The mean growth per cycle and the standard deviation values are given in each histogram.

**Figure 2:**
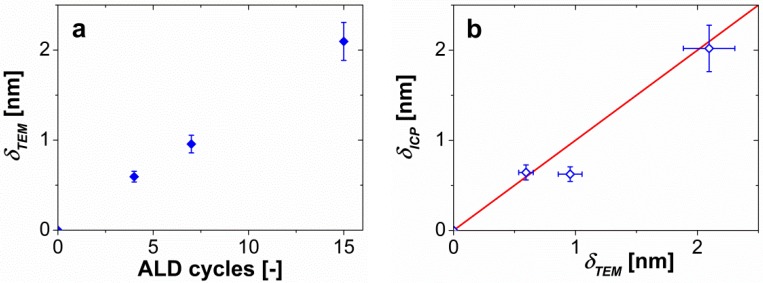
(**a**) Thickness of the alumina film, determined from TEM images, for the different number of cycles for a dosing sequence of 3.5–10–2.5–10 min. The error bars represent the 10% error assumed in the image analysis; (**b**) Comparison of the film thickness from TEM images and ICP-OES measurements for a density of alumina of 2500 kg/m^3^. The horizontal error bars represent the 10% error in the image analysis, while the vertical error bars show the error made in the calculation of the thickness, which accounts for the size of TiO_2_ particle, the experimental error of the ICP-OES device (3%) and the density of the alumina. The red line represents ideal agreement between the results.

Figure 1a–c shows TEM pictures, and the distribution of the growth per cycle (Figure 1d–f) for the 4, 7, and 15 ALD-cycle experiments, respectively. The values of the growth per cycle are similar to the results reported for ALD experiments at a few millibars and ~180 °C, *i.e.*, 0.1–0.2 nm. Although the histograms show some spread, from the TEM images we can see that the alumina coating on each particle is rather uniform and conformal. Figure 2a suggests a linear correlation between the number of cycles and the film thickness from TEM with a GPC of about 0.14–0.15 nm (Figure 2a), although it would require more data points to draw solid conclusions about linearity.

To support the results from the TEM images, we performed ICP-OES (Supplementary Information S5) on the samples to calculate the thickness of the alumina films with Equations (1) and (2), using a density of alumina of 2500 kg/m^3^ (Figure 2b). First, we observe that $x_{Al}$ for the samples coated with 4 and 7 cycles (Supplementary Information S5) are similar, while there is a clear difference in the film thickness observed from TEM pictures (Figure 1). We have not found a plausible explanation for the comparable values from ICP (Figure 2b). Besides these values for the 7-cycle experiment, which seem lower than expected, the other two values are in good agreement with those from the TEM (Supplementary Information S5). The error bars in Figure 2b represent the error in the measurements obtained from the propagation of the uncertainty (Supplementary Information S2).

In this study, we used relatively long pulsing and purging times to provide precursor molecules to the total surface area of powder, and to make sure that all non-absorbed reactants were removed. Nevertheless, if one is not carrying out a research study, but rather an industrial process, the initial load of powder inside the reactor may be increased and purging times reduced to find a proper compromise between production rate, reactant removal and operating costs.

### 3.2. Coating Experiments with Excess of Precursors at Ambient Conditions

After obtaining a reasonable value for the GPC in line with ALD literature, 0.14–0.15 nm, we studied what occurs when we feed an excess of precursor by increasing the dosage by a factor of 2, 4, and 6, compared to the base case dosing times (Figure 1b,e), and performed 7 cycles.

**Figure 3:**
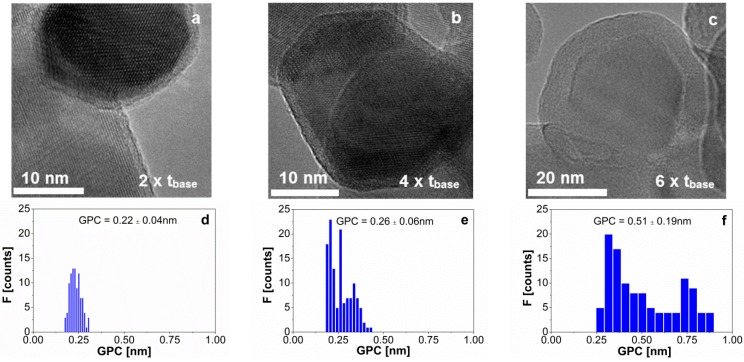
TEM image of the alumina film on TiO_2_ nanoparticles after 7 cycles when the dosing time of TMA per cycle is (**a**) 7 min; (**b**) 14 min; and (**c**) 21 min. The distribution of the GPC obtained from TEM images for a dosing time of TMA of (**d**) 7 min; (**e**) 14 min; and (**f**) 21 min. The mean and standard deviation values are shown in each histogram. The results in this figure can be compared with the ones of 7 cycles, with a dosing time of 3.5 min for TMA (Figure 1b,e), considered as the base case study.

**Figure 4:**
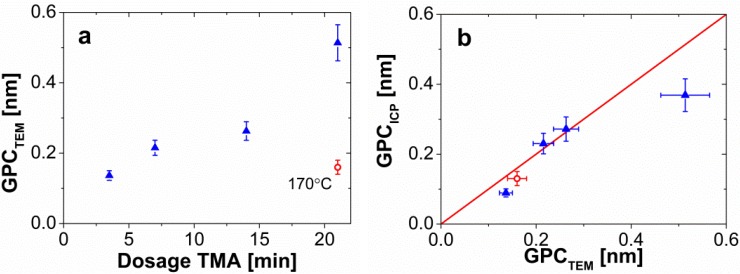
(**a**) GPC of the alumina film after 7 cycles with different dosing times of TMA per cycle (3.5, 7, 14, and 21 min). The red open circle represents an experiment carried out at 170 °C with a dosing time of TMA of 21 min. The error bars represent the error made in the image analysis of the TEM pictures, which is estimated to be around 10%. (**b**) Comparison of the GPC from TEM measurements and calculation from ICP-OES measurements for a density of alumina of 2500 kg/m^3^. The red open circle represents an experiment carried out at 170 °C with a dosing time of TMA of 21 min, for a density of the alumina of 3000 kg/m^3^. The horizontal error bars represent the 10% error in the image analysis, while the vertical error bars show the error made in the calculation of the thickness, which accounts for the size of TiO_2_ particle, the experimental error of the ICP-OES device (3%), and the density of the alumina. The red line represents the ideal agreement between the results.

Figure 3 shows the TEM pictures of the coating experiments with a dosage of TMA of 7 min (Figure 3a), 14 min (Figure 3b), and 21 min (Figure 3c) per cycle. The pulsing time of water was also increased accordingly for each of the experiments. Figure 3d–f gives the distribution of the growth per cycle for each of the experiments. The mean value and standard deviation, calculated from over 125 measurements (Supplementary Information S4), is shown in each histogram (Figure 3d–f). Although the films look uniform in the TEM pictures we assessed (Figure 3a–c), we observed a noticeable spread in the histograms compared to Figure 1. Nevertheless, if we calculate the normalized standard deviation (*i.e.*, standard deviation divided by the mean) for the growth per cycle, we observe similar relative spread of the data. The larger deposition rates obtained from Figure 3 compared to the base case study indicate the presence of a CVD type of growth, especially in the sample where TMA was fed for 21 min (Figure 3c,f). This suggests that part of the excess molecules adsorbs to the surface of the particles, and reacts during the subsequent reaction, which is a consequence of operating below the boiling temperature of TMA (~128 °C) and water. Figure 4a shows the increase of the growth per cycle with the dosing time of TMA.

We also calculated the thickness of the alumina films and the growth per cycle using the mass fraction of aluminium $x_{\text{Al}}$ from the ICP measurements. We observed a discrepancy between the measurements for the longest dosing time; this might be explained by the fact that CVD at these conditions leads to a different alumina density than the assumed 2500 kg/m^3^.

Although there seems to be a notable CVD-component present at these long pulse times—so we can no longer call the coating truly ALD—it is still possible to get conformal films of a reasonably controlled thickness. Nevertheless, the CVD component that occurs at ambient conditions can be avoided by increasing the reactor temperature.

### 3.3. Coating with Excess of Precursors at 1 bar and 170 °C

**Figure 5:**
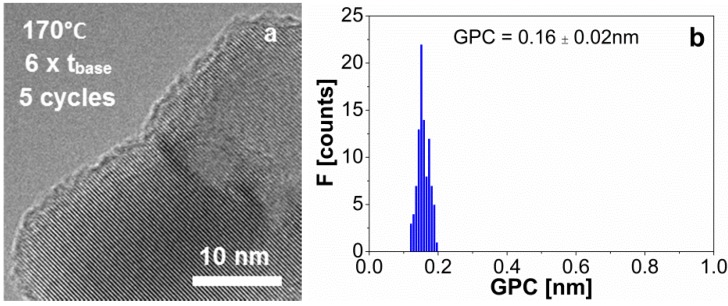
(**a**) TEM image of the alumina film on TiO_2_ nanoparticles after 5 ALD cycles for a dosing sequence of 21–10–15–10 min at 1 bar and 170 °C; (**b**) The distribution of the GPC obtained from TEM images for a dosing sequence of time of 21–10–15–10 min at 1 bar and 170 °C. The mean and standard deviation values are shown in the histogram.

To evaluate the influence of the reactor temperature on the physisorption of the precursor molecules in excess, we performed a coating experiment at higher temperature, *i.e.*, 170 °C. For that, we used the dosing times where we observed the strongest CVD component, *i.e.*, 21–10–15–10 min, and performed 5 cycles at 1 bar and 170 °C. We measured the thickness of the alumina films of about 20 particles using TEM (Figure 5a and Supplementary Information S4), and plotted a histogram of these values (Figure 5b). We measured a mass fraction of aluminum $x_{\text{Al}}$ in the sample of 0.044 with ICP-OES, which translated into a film thickness of 0.66 nm and a GPC of 0.13 nm (Figure 4a, red-open circular symbol). To calculate these values we used an alumina density of 3000 kg/m^3^, typical for Al_2_O_3_ ALD at 177 °C [36]. The results of film thickness and GPC obtained from elemental analysis agree with the values from the TEM images (Figure 5), and in general, with the GPC reported for similar ALD experiments at <1 mbar and ~180 °C, *i.e.*, 0.1–0.2 nm. The decrease in the GPC from 0.51 nm at room temperature to 0.16 nm at 170 °C (Figure 4a) indicates that the ALD reactions at these conditions, *i.e.*, 1 bar and 170 °C, are self-limiting even when both precursors are fed in large excess.

This work, as a first attempt to describe the ALD coating at room conditions, opens up the possibility for further research, such as that on the purging time. Being able to deposit controlled thin films at these conditions widens the potential use of alumina ALD to other heat sensitive materials, facilitating the coating process of particles at larger scales.

### 3.4. Alumina Film Density

Measuring the density of thin films on nanoparticles is not trivial with flat-substrate techniques such as ellipsometry. The low value of the density can indicate the presence of micropores or unreacted molecules in the film, although the results from Figure 2b and Figure 4b show a good agreement with the value of 2500 kg/m^3^ [36]. We performed N_2_-adsorption measurements using BET, and TGA as further analysis of the alumina films. We measured the surface area of three samples: P25 TiO_2_, the sample coated with 15 cycles and base case dosing times, and the sample coated with 7 cycles and a dosage of precursors six times larger than the base case study. The three samples showed similar adsorption and desorption isotherms (Supplementary Information S6), indicating that either there are no pores in the films, or that the pores have a small volume since there is no significant hysteresis in the isotherms. The surface area of the coated samples decreases compared to the uncoated nanoparticles (Supplementary Information S6), although this is explained by (i) the increase on the particle size due to the alumina coating, and (ii) by the decrease of the density of the core-shell particle, since the alumina film has a lower density than the P25 TiO_2_. These two factors do not provide evidence of pore formation in the alumina coating. Moreover, no pores are visible in the TEM images (Figure 2, Figure 4 and Supplementary Information S3 and S4), although that depends on the focus of the microscope. Both measurements suggest that the obtained films are not porous.

As an alternative explanation, the presence of unreacted water molecules or methyl groups inside the alumina coating could decrease the value of the film density. We performed TGA on the uncoated TiO_2_ and the samples coated with 4, 7 and 15 cycles to measure the mass loss when heating the samples from room temperature to 600 °C in air atmosphere. In this range of temperatures, we would observe first the evaporation of water molecules—both physisorbed and entrapped in the coating—and at higher temperatures the combustion of methyl groups. We found a total weight loss of <3.5%, which was mostly produced below 200 °C (Supplementary Information S7). This is attributed to water evaporation, part of it physisorbed molecules assumed from the analysis of the uncoated TiO_2_ sample, and part of it entrapped molecules within the coating. We compared the amount of water entrapped in the coating, with the amount of water dosed to the reactor, obtaining that the percentage of entrapped water is <0.1% (Supplementary Information S7). This suggests that the accumulation of unreacted water is negligible. The combustion of the unreacted methyl groups, which occurs above 400 °C, would produce an exothermic peak in the heat flow during the TGA and an increase in the SDTA (differential thermal analysis), which relates to the temperature of the sample and the chamber (Supplementary Information S7). At temperatures between 400 and 600 °C the mass loss recorded is <0.5% and the temperature difference between the sample and chamber is <0.5 °C for all the cases. We calculated the number of methyl groups of which combustion relates to this increase of temperature, resulting in <0.001% of the total amount of methyl groups being fed to the reactor during the whole experiment. This shows that there is not a substantial amount of unreacted water molecules or methyl groups to alter the properties of the alumina films. The low density of alumina we found—in agreement with the reported value for ALD at room temperature [36]—seems to be caused neither by porosity nor by the presence of unreacted species.

## 4. Conclusions

We demonstrated that ultrathin films of aluminium oxide can be deposited on particles using ALD at 27 °C and 1 bar in a well-mixed reactor, a fluidized bed. For that, we established that controlling the amount of precursor dosed to the reactor is crucial. For a set of experiments of 4, 7 and 15 cycles, we obtained a GPC of 0.14 nm for the defined dosing times. This result is comparable to the GPC of the experiments at ~170 °C and <1 mbar. We also calculated the thickness of the alumina films based on the elemental composition of aluminium in the samples, achieving a good agreement with the measured values from the TEM pictures. We increased the amount of the precursors by a factor of 2, 4 and 6 at constant flow, feed concentration and purging time, and performed 7 cycles. We observed that the thickness of the alumina films increased with the dosage of the precursors. A tentative explanation is that the unreacted molecules, which were fed in excess, accumulated on the surface of the particles, resulting in parasitic CVD reactions and higher GPC. In addition, we performed a coating experiment at 170 °C overdosing both precursors by a factor of 6 compared to the base case study, and obtained a GPC of 0.16 nm. This result agrees with the range of values of GPC reported for typical Al_2_O_3_ ALD reactions. We conclude that at room temperature and atmospheric pressure, this ALD process ceases to be self-limiting, but allows precise deposition of thin and uniform films by controlling the dosage of precursors. The obtained films are non-porous and do not contain noticeable amounts of unreacted species.

## Supplementary Materials

Supplementary materials can be accessed at: http://www.mdpi.com/1996-1944/8/3/1249/s1.
