# Supplementary material for: Gas-Phase Deposition of Ultrathin Aluminium Oxide Films on Nanoparticles at Ambient Conditions
Source: Materials (Basel). 2015 Mar 19;8(3):1249–63. doi: 10.3390/ma8031249 (PMC5455438; doi:10.3390/ma8031249)
Supplement: Supplementary file 1 [file materials-08-01249-s001.pdf]

Supplementary Information

S1. Particle Size Distribution TiO_2 P25 from TEM Images

We obtained the PSD (particle size distribution) of the TiO_2 P25 particles from the measurements of the diameter of 25 TEM pictures. We determined the distribution for each particle diameter, and plotted the PSD (Figures S1 and S2). From it, we calculated the Sauter mean diameter ($d_{3,2}$) using the equation below. The Sauter diameter accounts for the ration volume to surface area of the particles. The calculation of the alumina film thickness (Supplementary Information S2) is based on the surface area of the particles, then we consider that the $d_{3,2}$ is the most accurate representation of the mean particle size in order to calculate the film thickness. We also calculated the number-averaged diameter ($d_{1,0}$) from the transmission electron microscopy (TEM) measurement, observing the difference with the $d_{3,2}$. We consider that we do an error of 10% on the measurements of particle diameter from the TEM images ($\Delta d_{3,2}$ = 3 nm).

$$d_{3,2} = \frac{\sum n \cdot d_P^3}{\sum n \cdot d_P^2}$$

$$d_{1,0} = \frac{\sum d_P}{n}$$

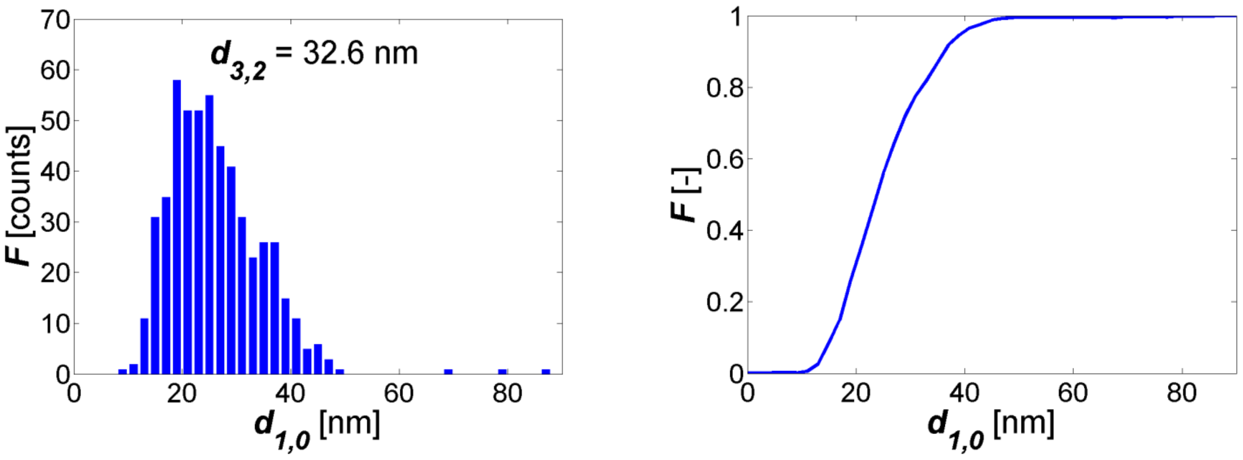

Figure S1. PSD of the P25 TiO_2 from TEM images. We measure the diameter in the TEM pictures, and from them we calculated the $d_{3,2}$ and plotted the distributions. We obtained an average $d_{3,2}$ = 32.7 nm.

Table S1. Determination of the PSD of TiO2 P25 from TEM images.

Sample	N TEM Pictures	N Particles	N Points	$d_{1,0}$ [nm]	$d_{3,2}$ [nm]
TiO_2 P25	25	>300	533	26.2	32.7

S2. Al_2O_3 Film Thickness Calculation

This calculation is based on the measurements of the mass fraction of aluminium (x_{Al}) on the coated samples, obtain from the ICP-OES. From this value, we calculate the volume of aluminium oxide per particle ($V_{\mathrm{Al2O3}}^{\mathrm{1P}}$) related to the mass fraction of aluminium. Finally, we calculate the thickness of the alumina film (δ_{ICP}) considering spherical particles. To account for the systematic error of the

measurements of the particle size ($\Delta d_{3,2}$) and aluminium mass fraction (Δx_{Al}), we perform the propagation of the uncertainty to calculate the error originated from the calculation of the film thickness ($\Delta\delta_{ICP}$).

S2.1. Equation of the Volume of Alumina Film

$$V_{Al2O3}^{TOTAL}\,[m^3] = x_{Al}[\frac{kg\ aluminium}{kg\ sample}] \cdot m_{sample}\,[kgsample] \cdot \frac{M_{Al2O3}[\frac{kg\ Al_2O_3}{kmol\ Al_2O_3}]}{M_{Al}[\frac{kg\ Al}{kmol\ Al}]} \cdot 0.5[\frac{kmol\ Al_2O_3}{kmol\ Al}] \cdot \frac{1}{\rho_{Al2O3}}[\frac{m^3_{Al2O3}}{kg_{Al2O3}}]$$

$$N_P = \frac{m_{TiO2}^{TOTAL}}{m_{TiO2}^{1p}} = \frac{m_{sample} - m_{Al2O3}}{\frac{\pi}{6} \cdot d_{3,2}^3 \cdot \rho_{TiO2}} = \frac{m_{sample} - x_{Al} \cdot m_{sample} \cdot \frac{M_{Al2O3}}{M_{Al}} \cdot 0.5}{\frac{\pi}{6} \cdot d_{3,2}^3 \cdot \rho_{TiO2}}$$

$$V_{Al2O3}^{1P}\,[m^3] = \frac{V_{Al2O3}^{TOTAL}}{N_P} = \frac{x_{Al} \cdot m_{sample} \cdot \frac{M_{Al2O3}}{M_{Al}} \cdot 0.5 \cdot \frac{1}{\rho_{Al2O3}}}{\frac{m_{sample} - x_{Al} \cdot m_{sample} \cdot \frac{M_{Al2O3}}{M_{Al}} \cdot 0.5}{\frac{\pi}{6} \cdot d_{3,2}^3 \cdot \rho_{TiO2}}}$$

$$= \frac{x_{Al} \cdot \frac{M_{Al2O3}}{M_{Al}} \cdot 0.5 \cdot \frac{\rho_{TiO2}}{\rho_{Al2O3}} \cdot \frac{\pi}{6} \cdot d_{3,2}^3}{1 - X_{Al} \cdot \frac{M_{Al2O3}}{M_{Al}} \cdot 0.5}$$

Then the volume of aluminium oxide per particle can be calculated with this equation:

$$V_{Al2O3}^{1P} = \frac{x_{Al} \cdot \frac{M_{Al2O3}}{M_{Al}} \cdot 0.5 \cdot \frac{\rho_{TiO2}}{\rho_{Al2O3}} \cdot \frac{\pi}{6} \cdot d_{3,2}^3}{1 - x_{Al} \cdot \frac{M_{Al2O3}}{M_{Al}} \cdot 0.5}$$

S2.2. Calculation of the Thickness of the Aluminium Oxide Film

$$V_{Al2O3}^{1P} = V_{Core-Shell}^{1P} - V_{TiO2}^{1P} = \frac{\pi}{6} \cdot \left(d_{3,2} + 2 \cdot \delta_{ICP}\right)^3 - \frac{\pi}{6} \cdot d_{3,2}^3$$

$$\delta_{ICP} = \frac{[(V_{Al2O3}^{1P} + \frac{\pi}{6} \cdot d_{3,2}^3) \cdot \frac{6}{\pi}]^{\frac{1}{3}} - d_{3,2}}{2}$$

$$\delta_{ICP} = \frac{(\frac{A \cdot x_{Al}}{1 - A \cdot X_{Al}} \cdot \frac{\rho_{TiO2}}{\rho_{Al2O3}} \cdot d_{3,2}^3 + d_{3,2}^3)^{\frac{1}{3}} - d_{3,2}}{2}$$

where:

$$A = \frac{M_{Al2O3}}{M_{Al}} \cdot 0.5$$

S2.3. Error Propagation Equation

$$\Delta\delta_{ICP} = \sqrt{\left(\frac{\partial\delta_{ICP}}{\partial x_{Al}}\cdot\Delta x_{Al}\right)^2 + \left(\frac{\partial\delta_{ICP}}{\partial d_{3,2}}\cdot\Delta d_{3,2}\right)^2 + \left(\frac{\partial\delta_{ICP}}{\partial \rho_{Al2O3}}\cdot\Delta \rho_{Al2O3}\right)^2}$$

$$\frac{\partial\delta_{ICP}}{\partial x_{Al}} = \frac{\frac{1}{6}\cdot\frac{\rho_{TiO2}}{\rho_{Al2O3}}\cdot d_{3,2}^3\cdot\frac{A}{(1-A\cdot x_{Al})^2}}{\left(\frac{A\cdot x_{Al}}{1-A\cdot x_{Al}}\cdot\frac{\rho_{TiO2}}{\rho_{Al2O3}}\cdot d_{3,2}^3 + d_{3,2}^3\right)^{\frac{2}{3}}}$$

$$\frac{\partial\delta_{ICP}}{\partial d_P} = \frac{\frac{1}{2}\cdot\left(\frac{A\cdot x_{Al}}{1-A\cdot x_{Al}}\cdot\frac{\rho_{TiO2}}{\rho_{Al2O3}}\cdot d_{3,2}^2 + d_{3,2}^2\right)}{\left(\frac{A\cdot x_{Al}}{1-A\cdot x_{Al}}\cdot\frac{\rho_{TiO2}}{\rho_{Al2O3}}\cdot d_{3,2}^3 + d_{3,2}^3\right)^{\frac{2}{3}}} - \frac{1}{2}$$

$$\frac{\partial\delta_{ICP}}{\partial \rho_{Al2O3}} = \frac{-\frac{1}{6}\cdot A\cdot X_{Al}\cdot\rho_{TiO2}\cdot d_P^3}{(1-A\cdot X_{Al})\cdot\rho_{Al2O3}^2\cdot\left(\frac{A\cdot X_{Al}}{1-A\cdot X_{Al}}\cdot\frac{\rho_{TiO2}}{\rho_{Al2O3}}\cdot d_P^3 + d_P^3\right)^{\frac{2}{3}}}$$

S3. TEM Pictures for Base Case Dosing Times after 4, 7, and 15 Cycles

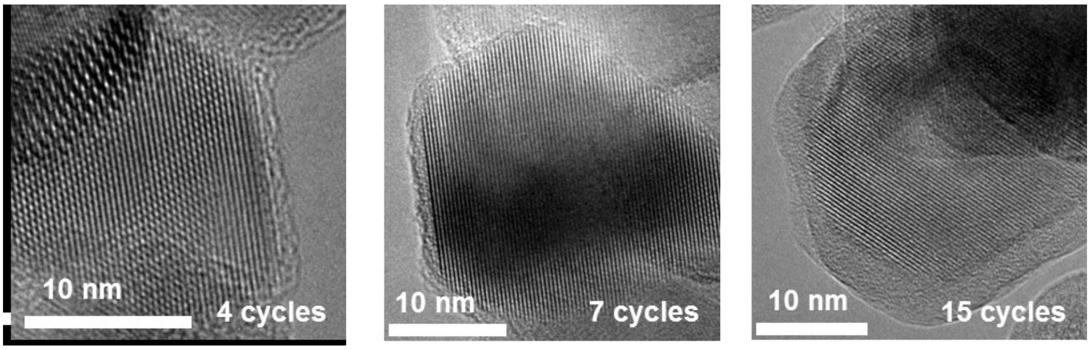

(**a**) (**b**) (**c**)

Figure S2. TEM images of the base case experiments with (**a**) four; (**b**) seven; and (**c**) 15 cycles.

Table S2. Determination of alumina film thickness from TEM images, with the standard deviation of the measurements.

Sample	N Pictures [-]	N Particles [-]	N Points [-]	δ_{TEM} [nm]	GPC [nm]
4 cycles	17	20	73	0.6 ± 0.1	0.15 ± 0.02
7 cycles	14	49	148	1.0 ± 0.2	0.14 ± 0.02
15 cycles	14	52	163	2.1 ± 0.6	0.14 ± 0.04

S4. TEM Pictures after 7 Cycles, Feeding the Precursors in Excess Compared to the Base Case

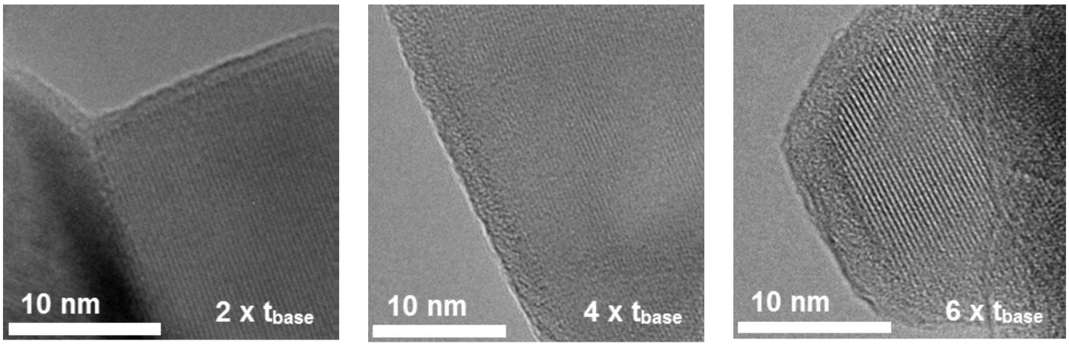

(a) (b) (c)

Figure S3. TEM images of the experiments where the precursors were dosing with twice, four and six times the dosing times of the base case study. (**a**) 2× t_{base}; (**b**) 4× t_{base}; (**c**) 6× t_{base}.

Table S3. Determination of alumina film thickness from TEM images, with the standard deviation of the measurements. Temperature is 27 °C unless indicated otherwise.

Sample	N Pictures [-]	N Particles [-]	N Points [-]	δ_{TEM} [nm]	GPC [nm]
$t_{base\ case}$	14	49	148	1.0 ± 0.2	0.14 ± 0.02
2 × $t_{base\ case}$	11	35	131	1.5 ± 0.3	0.22 ± 0.04
4 × $t_{base\ case}$	9	25	126	1.8 ± 0.4	0.26 ± 0.06
6 × $t_{base\ case}$	10	28	113	3.6 ± 1.3	0.51 ± 0.19
170 °C–6 × t_{base}	15	23	96	0.8 ± 0.1	0.16 ± 0.02

S5. ICP-OES Measurements

Table S4. Film thickness calculated from the ICP-OES measurements, and comparison with the results from the TEM measurements. The interval of confidence represents the error in the measurements as shown.

Sample	x_{Al} [-]	V^{1P}_{Al2O3} [m³]	δ_{ICP} [nm]	δ_{TEM} [nm]
4 cycles	0.036 ± 0.001	2.44 × 10^{-24}	0.64 ± 0.08	0.59 ± 0.06
7 cycles	0.035 ± 0.001	2.37 × 10^{-24}	0.62 ± 0.08	0.96 ± 0.10
15 cycles	0.105 ± 0.003	8.32 × 10^{-24}	2.02 ± 0.26	2.10 ± 0.21
2 × $t_{base\ case}$	0.086 ± 0.003	6.48 × 10^{-24}	1.61 ± 0.21	1.51 ± 0.15
4 × $t_{base\ case}$	0.100 ± 0.003	7.79 × 10^{-24}	1.90 ± 0.24	1.84 ± 0.18
6 × $t_{base\ case}$	0.131 ± 0.004	1.10 × 10^{-24}	2.58 ± 0.33	3.60 ± 0.36
170 °C–6 × t_{base}	0.044 ± 0.001	2.30 × 10^{-24}	0.66 ± 0.08	0.78 ± 0.08

S6. BET Measurements of the Porosity

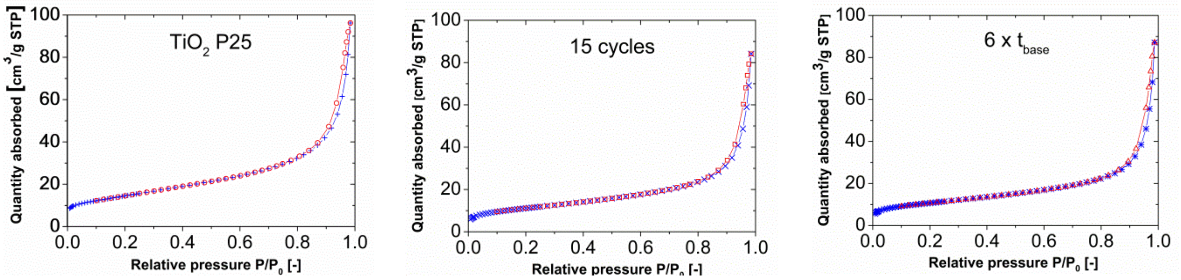

Figure S4. BET isotherms for the uncoated TiO_2 (**left**), sample coated with 15 cycles (**middle**), and sample coated with a dosage of precursors six times larger than the base case study (**right**).

Table S5. Comparison of the surface area measured with BET and calculated.

Samples	δ_{TEM} [nm]	SA_{BET} [m²/g]	ρ_{eq} [kg/m³]	SA_{calc} [m²/g]
Un-coated TiO2	-	52.4 ± 0.1	-	-
15 cycles	2.1 ± 0.7	39.9 ± 0.1	3683 ± 141	44.1 ± 4.6
$6 \times t_{dosing}$	3.7 ± 1.5	38.2 ± 0.1	3422 ± 213	43.7 ± 5.6

We use the values of the particle size measured ($d_{3,2}$ = 33 ± 3 nm), density of TiO_2 (ρ_{TiO2} = 4200 kg/m³) and density of the alumina film (ρ_{Al2O3} = 2500 kg/m³) to compare the measured surface area (SA_{BET}) with the calculated value (SA_{calc}). We calculated the interval of confidence of the measurement by calculating the propagation of the uncertainty. These are the equations used:

$$SA_{calc} = \frac{6}{(d_{3,2} + 2 \cdot \delta_{TEM}) \cdot \rho_{eq}}$$

$$\rho_{eq} = \frac{V_P}{V_{TOTAL}} \cdot \rho_{TiO2} + \frac{V_{Al2O3}}{V_{TOTAL}} \cdot \rho_{Al2O3} = \frac{{d_{3,2}}^3}{(d_{3,2} + 2 \cdot \delta_{TEM})^3} \cdot \rho_{TiO2} + \frac{(d_{3,2} + 2 \cdot \delta_{TEM})^3 - {d_{3,2}}^3}{(d_{3,2} + 2 \cdot \delta_{TEM})^3} \cdot \rho_{Al2O3}$$

$$\Delta\rho_{eq} = \sqrt{\left(\frac{\partial \rho_{eq}}{\partial d_{3,2}} \cdot \Delta d_{3,2}\right)^2 + \left(\frac{\partial \rho_{eq}}{\partial \delta_{TEM}} \cdot \Delta\delta_{TEM}\right)^2}$$

$$\Delta SA_{calc} = \sqrt{\left(\frac{\partial SA_{calc}}{\partial d_{3,2}} \cdot \Delta d_{3,2}\right)^2 + \left(\frac{\partial SA_{calc}}{\partial \delta_{TEM}} \cdot \Delta\delta_{TEM}\right)^2 + \left(\frac{\partial SA_{calc}}{\partial \rho_{eq}} \cdot \Delta\rho_{eq}\right)^2}$$

S7. Thermo Gravimetric Analysis

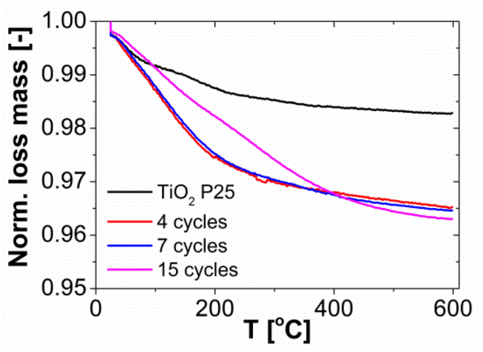

ALD cycles	P25 TiO_2	4	7	15
mass initial [mg]	7.00	5.20	8.60	9.50
mass end [mg]	6.88	5.02	8.30	9.15
Δmass [mg]	0.12	0.18	0.30	0.35
N H_2O loss [mol]	0	3.4×10^{-6}	1.0×10^{-5}	1.3×10^{-5}
N H_2O dosed [mol]	0	0.007	0.012	0.025
H_2O desorbed [%]	0	0.05	0.09	0.05
Δmass >400 °C [mg]	0.01	0.01	0.03	0.05
Δmass >400 °C [%]	0.1	0.3	0.3	0.5

Figure S5. (**left**) Mass loss profile for the uncoated TiO2 and the samples coated with four, seven and 15 cycles; (**right**) Calculation of the percentage of the water molecules fed to the system compared to the amount of water released during the TGA analysis.

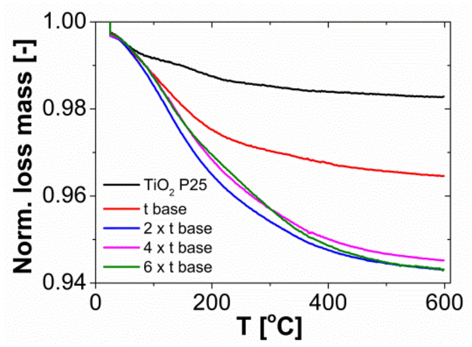

Dosage TMA	P25 TiO_2	t_{base}	2× t_{base}	6× t_{base}
mass initial [mg]	7.00	8.60	10.90	8.60
mass end [mg]	6.88	8.30	10.28	8.11
Δmass [mg]	0.12	0.30	0.62	0.49
N H_2O loss [mol]	0	1.0×10^{-5}	2.8×10^{-5}	2.0×10^{-5}
N H_2O dosed [mol]	0	1.2×10^{-2}	2.4×10^{-2}	7.1×10^{-2}
H_2O desorbed [%]	0	0.09	0.12	0.03
Δmass >400 °C [mg]	0.0075	0.025	0.048	0.047
Δmass >400 °C [%]	0.11	0.30	0.44	0.54

Figure S6. (**left**) Mass loss profile for the uncoated TiO2 and the samples coated with an excess of precursors of two and six times that of the base case study; (**right**) Calculation of the percentage of the water molecules fed to the system compared to the amount of water released during the TGA analysis.

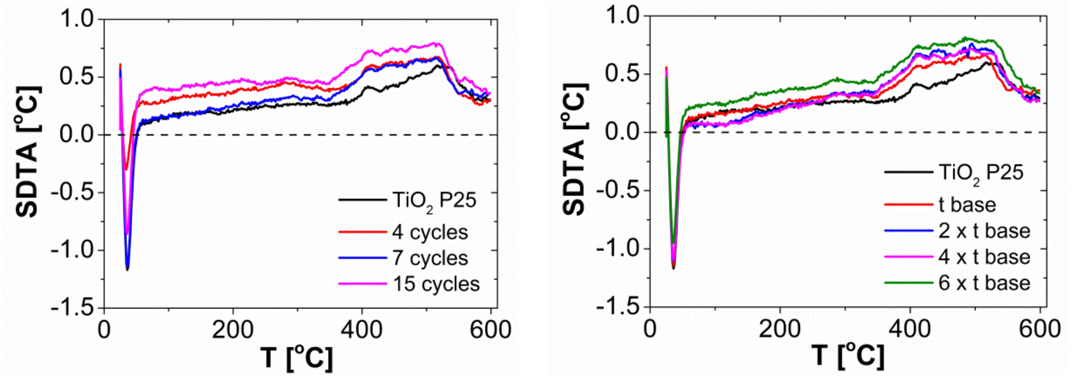

Figure S7. Differential Thermal Analysis (SDTA) from the TGA, for the base case study (**left**); and when the precursors where fed in excess (**right**).
